# The Art of Framework Construction: Core–Shell Structured Micro-Energetic Materials

**DOI:** 10.3390/molecules26185650

**Published:** 2021-09-17

**Authors:** Binghui Duan, Jiankang Li, Hongchang Mo, Xianming Lu, Minghui Xu, Bozhou Wang, Ning Liu

**Affiliations:** 1 Department of Energetic Materials Science and Technology, Xi’an Modern Chemistry Research Institute, Xi’an 710065, China; duanbinghui@126.com (B.D.); hongchangmo@163.com (H.M.); luxianming1220@126.com (X.L.); mhuixu@163.com (M.X.); wbz600@163.com (B.W.); 2 State Key Laboratory of Fluorine & Nitrogen Chemicals, Xi’an Modern Chemistry Research Institute, Xi’an 710065, China

**Keywords:** core–shell structure, micro-energetic materials, preparation, insensitivity, formation mechanism

## Abstract

Weak interfacial interactions remain a bottleneck for composite materials due to their weakened performance and restricted applications. The development of core–shell engineering shed light on the preparation of compact and intact composites with improved interfacial interactions. This review addresses how core–shell engineering has been applied to energetic materials, with emphasis upon how micro-energetic materials, the most widely used particles in the military field, can be generated in a rational way. The preparation methods of core–shell structured explosives (CSEs) developed in the past few decades are summarized herein. Case studies on polymer-, explosive- and novel materials-based CSEs are presented in terms of their compositions and physical properties (e.g., thermal stability, mechanical properties and sensitivity). The mechanisms behind the dramatic and divergent properties of CSEs are also clarified. A glimpse of the future in this area is given to show the potential for CSEs and some suggestions regarding the future research directions are proposed.

## 1. Introduction

In materials science, energetic materials (EMs) capable of storing and releasing large amounts of chemical energy are widely used in military and civilian areas [1,2]. Micro-energetic materials whose particle sizes range between several micrometers and hundreds of micrometers are mostly engaged in propellants and large-scaled explosives. High energy and safety are the greatest concerns and have always been an inherent contradiction of energetic materials because high energy levels are mostly achieved at the expense of stability [3,4,5]. Therefore, extensive work has been done to balance the stringent requirements for power and sensitivity. Because the synthesis of novel insensitive energetic compounds develops slowly, current techniques for the desensitization of high explosives mainly include mediating particle size and morphology, improving crystal quality by recrystallization, exploring energetic cocrystals and preparing the polymer bonded explosives (PBXs) [6,7,8,9,10,11,12]. Among these strategies, techniques based on PBXs are considered most efficient to tune the performance of explosives. Nevertheless, due to the differences of molecular structure and polarity between explosives and polymer binders, the composites suffer from poor compatibility and weak interfacial interaction sometimes, resulting in poor mechanical properties [13,14,15]. The introduction of the core–shell strategy to PBXs has provided an elegant method to achieve explosives with better integrated performance.

**Figure 1:**
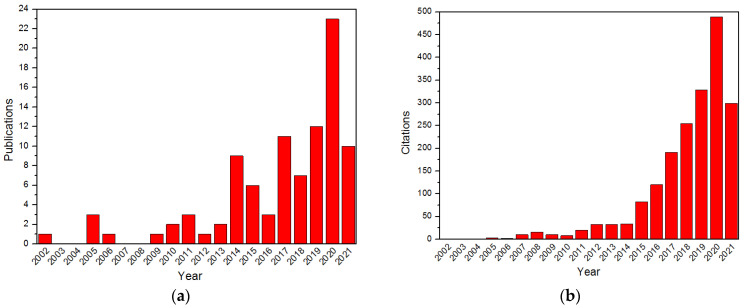
The number of publications (**a**) and citations (**b**) per year on CSEs (source Web of Science, September 2021).

As an important branch of coating, the design and preparation of core–shell structures have attracted much attention recently due to their potential applications [16,17,18,19]. Over the last decade, the number of publications and citations in terms of core–shell structured explosives (CSEs) has increased significantly, as shown in Figure 1. The types of core–shell energetic composites and the preparation of controllable core–shell structures have made great advances. In general, CSEs consist of one kind of energetic material that can be regarded as the “core” and another coating component which can be seen as the “shell”. The core components are wrapped by shell materials through chemical bonds or intermolecular interactions. The core–shell structure possesses the advantages of both core and shell materials and can also regulate the assembly and contents of core and shell according to the application requirements [20,21,22]. Therefore, CSEs have a wider application prospect than a single explosive. It is expected that an integral CSE will have the high energy density of the core and the insensitivity of the shell. Compared to the rapid development of core–shell engineering in catalysis, electronics, photoluminescence and biomedicine field [23,24,25,26,27,28,29], the application of core–shell techniques in energetic materials has had a slow start. There are two major problems responsible for this; one is that the sensitivity and mechanically fragile nature of energetic crystals increase the process handling difficulty, the other is that the smooth and chemically inert surface of explosive particles leads to the weak interfacial interactions between particles [30,31,32]. Fortunately, recent developments in core–shell engineering have significantly enhanced our understanding of the formation mechanism of core–shell structures and some pioneering researchers have shown that energetic core–shell structures can be realized based on hydrogen bonding and π–π conjunction [33,34,35].

In essence, most pure explosives possess high sensitivity. Coating insensitive components on the explosive particles suppresses the formation of hot-spots and reduces the risk of accidental explosion [36,37,38]. Application of the core–shell strategy can achieve a complete surface coverage with minimal use of coating materials to ensure energy performance and can be extended to different types of EMs with simple and mild methods [39,40]. In general, polymers or insensitive explosives are selected as shell components in CSE formulations to improve their mechanical strength and safety properties. It is known that the key indicators to evaluate a core–shell material are the degree of coverage and the adhesion force between core and shell materials. Various novel components [41,42,43,44,45] (e.g., dopamine and graphene) and preparation techniques (e.g., in situ polymerization and the emulsion method) have been developed to construct core–shell structures. The novel components and advanced preparation methods have proved to be effective in tailoring the properties of CSEs.

Although extensive data have been published in terms of preparation and characterization of CSEs, a comprehensive summary of the advancements in preparation methods and promising CSEs has not been presented to date. This contribution will focus upon CSEs with special emphasis upon the following: preparation methods, compositions, fundamental properties and the formation mechanism of core–shell structures. Especially, the characteristics of different preparation methods are analyzed, and the reasons for the improvements in the properties are explained. This review aims to deepen the understanding of core–shell structure and promote the evolution of CSEs.

## 2. Preparation Methods for CSEs

### 2.1. Water Suspension Method

**Figure 2:**
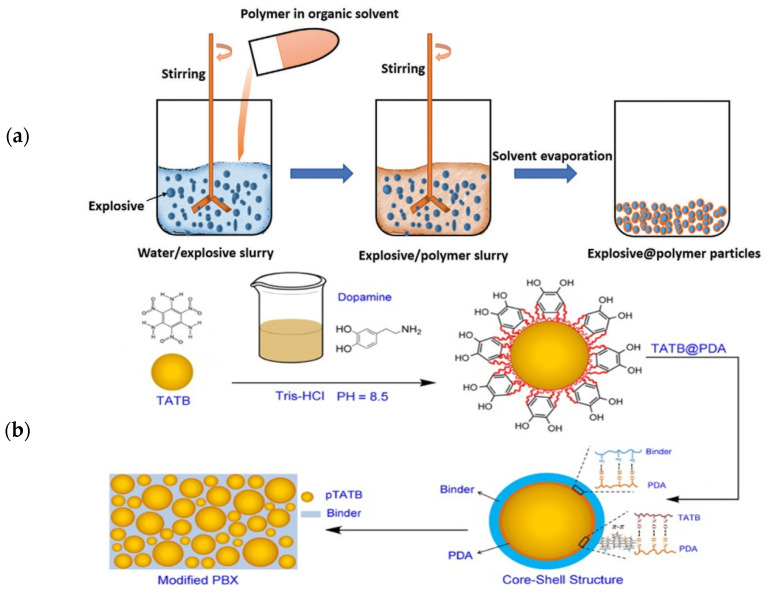

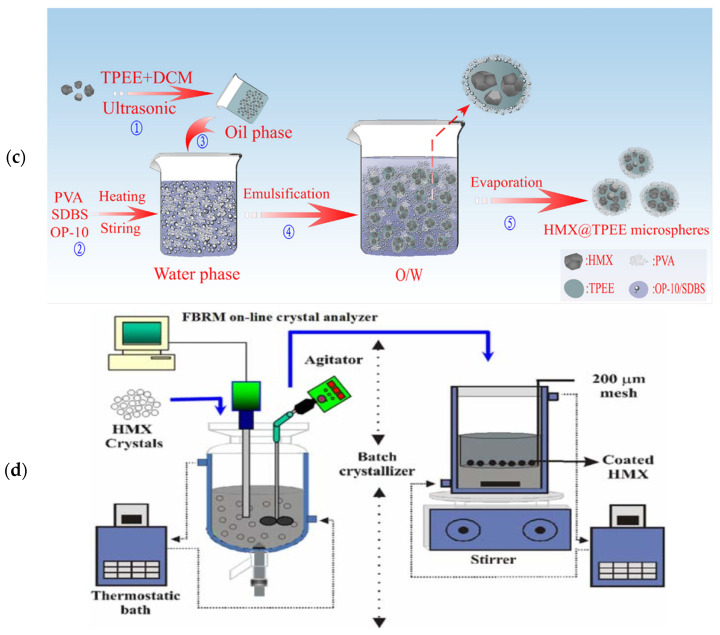
Schematic illustrations of four fabrication methods for CSEs: (**a**) water suspension method; (**b**) in situ polymerization method; (**c**) emulsion technique; (**d**) crystallization coating technique. (**a**) Reproduced with permission from [17], copyright 2020, Wiley-VCH. (**b**) Reproduced with permission from [33], copyright 2017, Royal Society of Chemistry. (**c**) Reproduced with permission from [76], copyright 2017, Elsevier. (**d**) Reproduced with permission from [82], copyright 2007, Wiley-VCH.

The water suspension method together with solvent–nonsolvent mixing is the most common practice to prepare CSEs. In this method, a certain amount of explosive is added to pure water to obtain uniform dispersion under stirring which is called an explosive suspension. Then, the polymer solution with the polymer dissolved in an organic solvent is added dropwise to the explosive suspension. The solvent is removed under heating and a slight vacuum. During the solvent evaporation process, the explosive core is coated and forms an agglomeration of explosive@polymer particles [46,47,48]. The process of water suspension to prepare CSEs is depicted in Figure 2a.

Extensive work has been done to investigate the influence of experimental parameters (e.g., the agitation rate, heating rate and temperature) on the quality of the composite. The variables including the size, shape, coating uniformity and integrity are the main areas of concern. Kasprzyk and Bell [49] investigated the effects of agitation speed, reactor temperature and air sweep rate on the production of PBX 9501 molding powder using the water suspension method. An increase in agitation speed leads to smaller particle size and higher bulk density. It is interesting to find that the shape of the molding powder, which is considered to determine the bulk density to a large extent, changes as the agglomerates grow. However, the mechanical strength of the molding powder has a slight dependence on the particle size and bulk density. An et al. [50] successfully prepared a cyclotrimethylenetrinitramine (RDX) composite with 2,4,6-trinitrotoluene (TNT) and an energetic material (HP-1) as the shell material. They stated that there existed optimal mass ratio of HP-1 to TNT, stirring speed, and cooling rate to achieve the best coating effect. The authors proposed a coating mechanism: TNT and HP-1 can form a liquid composite, adhere onto the RDX particles, and then grow on the surface of RDX with the decrease in temperature, which could account for the necessity of the process parameters chosen.

**Figure 3:**
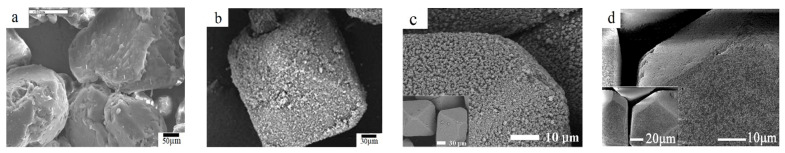

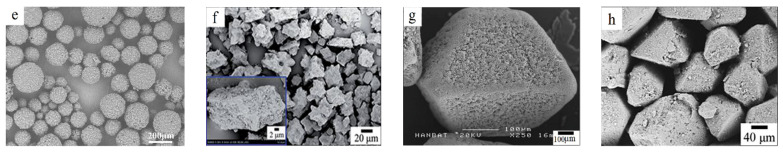
SEM images of some typical CSEs fabricated by different methods: (**a**) RDX@TNT/HP-1 via water suspension method; (**b**) CL-20@TATB via water suspension method; (**c**) HMX@MF via in situ polymerization technique; (**d**) HMX@PDA via in situ polymerization technique; (**e**) HMX@TPEE via emulsion solvent evaporation method; (**f**) HMX@TATB via spray drying method; (**g**) HMX@NTO via crystallization coating method; (**h**) HMX@TATB via ultrasonic method. (**a**) Reproduced with permission from [50], copyright 2009, Wiley-VCH. (**b**) Reproduced with permission from [51], copyright 2013, Wiley-VCH. (**c**) Reproduced with permission from [60], copyright 2015, Elsevier. (**d**) Reproduced with permission from [64], copyright 2017, Elsevier. (**e**) Reproduced with permission from [76], copyright 2017, Elsevier. (**f**) Reproduced with permission from [85], copyright 2015, Royal Society of Chemistry. (**g**) Reproduced with permission from [81], copyright 2011, American Chemical Society. (**h**) Reproduced with permission from [93], copyright 2014, Elsevier.

Yang et al. [27] offered up unique insights into the effects of crystal quality and morphology on the mechanical properties of 2,6-diamino-3,5-dinitropyrazine-1-oxide (LLM-105)@fluoropolymer composites. The authors revealed that the improved mechanical strength of the composite could be attributed to its neat spherical morphology and rough surface. However, recrystallized LLM-105 with high quality led to reduced tensile strength of the core–shell composite. A possible explanation is that the rough surface with anchor points and a high-quality internal structure are of vital importance to obtain a desirable core–shell structure as the anchor points enhance the interaction between the core surface and the polymer shell. In addition, 2,4,6,8,10,12-hexanitro-2,4,6,8,10,12-hexaazaisowurtzitane (CL-20)@1,3,5-triamino-2,4,6-trinitrobenzene (TATB) core–shell composite [51] was also prepared by the research group through a water suspension process. Surfactants, such as polyvinyl alcohol (PVA) and Tween-20, were used to improve the wettability of CL-20 and ensure the dispersion of TATB particles. The morphology of the prepared CL-20@TATB is shown in Figure 3b. The crystal form of CL-20 in the core–shell composite maintained the optimum ɛ form. Compared with a physical mixture of CL-20 and TATB, the core–shell composite featured compact coating and high coverage, while most TATB and CL-20 molecules exist independently in the mixture.

Water suspension method features moderate processing condition and versatility for most coating systems. The simple and straightforward physical protocol protects the core from significant changes, resulting in particles with well quality and shape. The weight ratio of shell to core and agitation speed should be considered with a view to the quality of core–shell composite. One should note that for small grains of explosives, especially below the micrometer scale, the water suspension method is not appropriate to construct core–shell structure since the aggregation of particles has frequently been reported.

### 2.2. In Situ Polymerization

The in situ polymerization method, extensively applied to microencapsulation and surface coating, is a direct chemical route to achieve high coverage and firm adhesion force between the explosive and coating materials [35,52,53,54,55]. The in situ polymerization process involves two steps: first, the monomers of coating shell are absorbed to the surface of core explosives through preferential interactions, they then polymerize on the core surface in situ under certain reaction conditions. Thus, in situ polymerization renders the composite with higher adhesion force and shell strength than a simple physical mixing process. Up to now, there are almost twenty candidates available as shell materials. The core–shell products and their features prepared by in situ polymerization method have been summarized in Table 1. It can be seen that most of the composites can achieve high coverage and uniform coating with shell contents of no more than 5%.

Polydopamine (PDA) coating has attracted much attention in the past decade due to the strong adhesive attachment of PDA to various surfaces under ambient conditions [20,39,56]. Gong, He and Lin conducted a series of studies [34,35,52,54,57] on in situ polymerization of dopamine for typical explosive crystals including cyclotetramethylenetetranitramine (HMX), TATB and CL-20. The schematic for the preparation of core–shell structured TATB composites and the supposed interactions between TATB, PDA and fluoropolymer are illustrated in Figure 2b. It was found that the surface of the explosive was wrapped by the PDA layer completely and the composites displayed an evident surface color change from yellow to brown. He et al. [55] proposed a “grafting-from” route to construct TATB-based core@double-shell (CDS) composites by in situ grafting hyperbranched polymers (HBPs) on the PDA surface. It has been demonstrated that PDA and HBP shells have synergism in enhanced mechanical properties and improved interfacial adhesion. Another similar work was reported by Zeng et al. [58] in which the PDA layer served as interfacial layer of TATB particles with three polymer binders: glycidyl azide polymer (GAP), polyethylene glycol (PEG) and polytetramethylene ether glycol (PTMEG), leading to outstanding mechanical properties and good compatibility between the composites and binders in PBXs.

Melamine–formaldehyde (MF) resins, popularly used as a coating shell for the fabrication of core–shell composites, are synthesized by the polycondensation reaction of melamine and formaldehyde molecules [59]. Yang et al. prepared RDX, HMX and CL-20-based CSEs [60] via in situ polymerization of MF resins on the explosive surface. It was found that the whole surface of the energetic core was completely and uniformly covered with 3% MF resins. A core-etching test was performed for CL-20@MF CSE and its physical mixture with the same component amounts. In striking contrast to the mixture, the MF resin shell of CSE was well connected and maintained fairly high mechanical resistance under vigorous stirring. Urea formaldehyde (UF) resin [61] and melamine urea formaldehyde (MUF) resin [62] were also utilized to fabricate HMX and CL-20. Studies indicate that this kind of resin with mild reaction conditions and desirable mechanical and stability performance are accommodative in the framework of CSEs. One should note that the addition of PVA plays an essential role during the process in that PVA increases the surface interactions between the resins and the explosive crystals and restricts the self-agglomeration of shell materials. Zhang et al. [63] produced HMX@polyaniline (PANI) CSEs using the in situ polymerization method. Before polymerization reaction, (3-aminopropyl)trimethoxysilane (APTES) was adopted to modify the HMX in order to increase the amino groups on the its surface. APTES acts similar to PDA as a linkage to enhance the adhesion between explosive particles and polymers. The scanning electron microscopy (SEM) images demonstrated that HMX particles were uniformly coated by a layer of polymer. Although there are limited monomers for in situ polymerization in mild conditions without destroying the structures of explosive crystals, this approach shows potential in designing and fabricating novel composites with integrated performance.

**Table 1:** Characteristics of the products prepared by in situ polymerization method.

Product	Size of Core [Diameter, μm]	Thickness of Shell [nm]	Shell Content [wt%]	Degree of Coverage	Feature	Comments	Contributor
TATB@PDA	14	NA	1.5	Close to 100%	Homogeneous PDA coating, coupled with obvious surface color change.		[52]
HMX(HNIW)@UF resin	20 (5–40)	NA	4.8 (4.3)	98.1% (95.3%)	Compact coating, without shrinks or bubbles.		[61]
TATB@HBP	20	NA	1.5	NA	Intact coating, rough surface.		[55]
CL-20/HMX/RDX@MF resin	120/120/60	1–2 μm	3.0	99.2%/98.7%/93.1%	Compact and uniform coating, slight agglomeration.	The reaction time should be well controlled to reduce self-agglomeration of shell material.	[60]
HMX@PDA	22	100	2.1	NA	Dense coating with PDA depositing layer-by-layer on the HMX crystal.		[64]
HMX@MPNs ^1^	91	50	3.4	NA	The composite particles have more textured surface with negligible wrinkles or holes.	Increasing the coating times may be an effective way to improve the compactness and mechanical strength through sequential layer deposition.	[65]
HMX@HPW ^1^@PDA	47	NA	NA	NA	A novel litchi-like core@double shell structure.		[66]
ε-CL-20@PDA	60	NA	1.6	NA	The composite particles have polyhedron shapes with uniform and compact coating.		[57]
HMX@BAMO-THF	23	NA	1.5	NA	The particle size distribution was relatively uniform, and the crystal quality greatly improved after coating.		[67]
HMX/rGO/G ^1^	10	NA	2.0	NA	Spherical morphology of the composite, different from angular HMX.		[43]
HMX@TATB@PDA	149.1	50–80	NA	NA	Uniform and porous surface.		[35]
LLM-105@PDA@HBPU ^1^	50,20,5	NA	1.0	NA	A layer of plicate characteristics with nanoscale protuberances on the shell.		[12]
HMX@PANI	5–40	NA	3.1	NA	Compact coating, few agglomerations and larger roughness after coating.		[63]
CL-20/HMX/RDX@MUF resin	10	NA	5.0	NA	Spheroidized structure with dense and smooth surface.	Core–shell structured composites with high quality can be achieved.	[62]
HMX@TATB	<250	NA	42.5	NA	HMX core has been jacketed with a layer of TATB particles.		[68]
CL-20@TATB	98	NA	NA	NA	Uniform coating.		[69]
NBTTP ^1^@PDA/GO	5–15	NA	2.0	NA	Regular color and particle size of all the samples.		[70]
HMX@Polyurethane	25.59	NA	NA	NA	More uniform, complete and smooth surface than virgin HMX particles.		[71]
HMX@HTPB/GAP/BAMO-THF	NA	NA	5.0	NA	Almost uniform coating.		[72]

^1^ Abbreviations: MPNs: Metal–phenolic networks; HPW: High melting point paraffin wax; rGO: reduced graphene oxide; G: graphene; HBPU: Hyperbranched polyurethane; NBTTP: Tetranitro-benzopyridotetraazapentalene.

### 2.3. Emulsion Method

The emulsion method can fabricate spherical core–shell composites and improve the homogeneity of particles. In this method, an oil phase (O) is obtained by dissolving polymer shell material into a suitable organic solvent and dispersing explosive in the polymer solution subsequently. The water phase (W) is derived from mixing emulsifier and stabilizer solution. Then, the two phases are blended together, and the composite particles are filtered after evaporating the solvent. The emulsion droplets remain stable and dispersive, preventing the coating particles from aggregation [19,73,74,75]. The emulsion polymerization is easy to operate. Most of the medium in the polymerization process is water, which avoids the trouble of using high solvents and their recovery, ensures the safety of the experiment to the greatest extent and reduces the possibility of pollution and fire.

Li et al. used the emulsion solvent evaporation (ESV) method to fabricate HMX with a thermoplastic polyester-ether elastomer (TPEE) coating as shown in Figure 2c [76]. Stabilizer PVA, emulsifier sodium dodecylbenzene sulfonate (SDBS) and nonaphenol polyethyleneoxy ether (OP-10) were adopted to form a stable emulsion. It was found that the HMX@TPEE composites exhibited a honeycomb-like core–shell structure and narrow size distribution (Figure 3e). The formation of a stable O/W emulsion and the precipitation of TPEE polymers on the explosive surface were supposed to be key elements in the formulation of well-shaped spherical particles. Liao et al. prepared CL-20@waterborne polyurethane grafted styrene and acrylonitrile copolymer (WPU-g-SAN) core–shell composites [77] via an emulsion polymerization method. The amorphous structure of WPU-g-SAN copolymer with asymmetrical styrene and acrylonitrile units favors the coating effect. X-ray photoelectron spectroscopy (XPS) and SEM analysis confirmed a well-shaped core–shell structure.

With the development of self-assembly and membrane techniques, the combination of emulsion and the newly developed methods have become popular trend to obtain desirable CSEs. Wang et al. [78] prepared CL-20/cellulose acetate butyrate (CAB) CSE through the premix membrane emulsification method. The coarse emulsion was pushed through a membrane to produce a homogeneous emulsion solution under pressure. It is interesting to find that the morphological structure of the composites changes from dumbbell shape to spherical with an increase in the CAB content. SEM and X-ray powder diffraction (XRD) analyses showed regular solid spherical particles with a smooth surface and dense coating layer. Jia et al. [79] reported a strategy utilizing poly methyl metharylate (PMMA)-PVA as the shell material and CL-20, HMX and RDX as cores for molecular collaborative self-assembly. The authors revealed that a honeycomb structure of the core–shell composite was formed based on hydrogen bonding between the explosive and the PMMA-PVA emulsion, which can promote self-assembly of the particles.

Emulsion method shows great potential for scale-up production due to its good operability, simplicity, and profitable processing performance. This approach is more mature in fabricating small particles of core explosives at several micrometer levels. One drawback is that emulsion process requires various chemical additives, such as monomers, emulsifier, stabilizer and initiator, which may integrate with shell materials, thus increasing the risk of chemical incompatibility among the components.

### 2.4. Crystallization Coating Method

Crystallization technique is a convenient and flexible method to tune the crystal size and shape in the dissolution and cooling process. The development of the crystallization coating method in the energetic material community has provided an alternative method to achieve EMs within the specifications of desirable crystal morphology and narrow crystal size distribution. The appearance and sensitivity performance can be optimized by selecting the right operating conditions, such as the solvent type, the concentration ratio of shell to core, stirring speed and the degree of sub-cooling [80,81].

Kim et al. [82] reported a novel HMX@3-nitro-1,2,4-triazole-5-one (NTO) CSE by embedding an NTO shell onto the surface of an HMX core. The crystallization coating process is shown in Figure 2d. The formation of the core–shell structure in the crystallization coating process was shown to be an agglomeration and crystal growth mechanism. In situ measurement of agglomeration tests were carried out using an Lasentec particle analyzer with a focused beam reflectance measurement (FBRM) control interface. The tests verified the proposed mechanism that the surface nucleation occurred first, and then the fine agglomerates grew on the surface of the HMX seed. During the agglomeration process, supersaturation was the most important parameter in producing agglomerated particles. The kinetics of agglomeration revealed that a relative high cooling rate facilitated supersaturation, leading to a uniform coating on the HMX surface (Figure 3g).

### 2.5. Spray Drying Method

Spray drying is a well-established, cost-effective method for producing microparticles [83,84,85]. Recently, the spray drying method has been introduced to the energetic materials field including fabrication of CSEs, cocrystals and explosive recrystallization due to the simplicity of the “all-liquid” precursor and one-step crystallization and formulation operation [86,87]. Furthermore, such an effective and reliable synthesis strategy is potential and advantageous for the quick and large-scale production of EMs.

**Figure 4:**
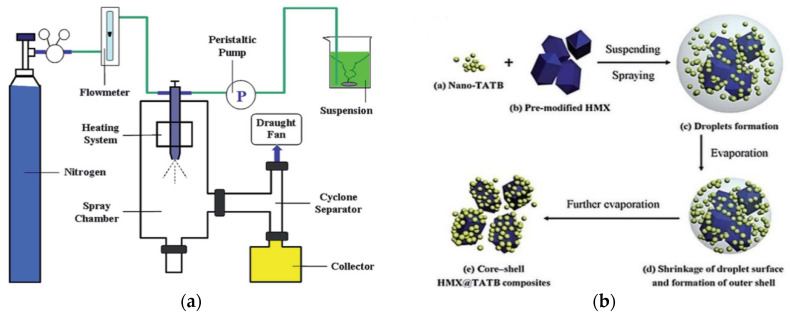
(**a**) Experimental apparatus of spray drying technique; (**b**) formation mechanism of HMX@TATB composite. (**a**,**b**) Reproduced with permission from [85], copyright 2015, the Royal Society of Chemistry.

Ma et al. [88] prepared HMX@TATB CSEs through a spray drying process as shown in Figure 4a. The surface of HMX@TATB core–shell micro-composites become rough because the outer TATB layer possessed grainy characteristics (Figure 3f). The thickness of the TATB shell is about 2 μm with a fairly high utilization of shell materials. The crystalline phase of *β*-HMX remained unchanged during the spray drying process due to the mild coating conditions. A formation schematic of the core–shell HMX@TATB composites is shown in Figure 4b. An aqueous dispersion containing TATB nanoparticles and pre-modified HMX microparticles is atomized into droplets, followed by solvent evaporation with a hot gas. Once the droplets contact the hot gas, TATB is rapidly precipitated and coated on the surface of HMX particles. Yang et al. [89] prepared a core–shell structure with fluoroelastomer (F_2602_) coated on 1,1-diamino-2,2-dinitroethylene (FOX-7) using a spray drying strategy. The mean particle size of the composites was reduced remarkably from 39.72 μm of raw FOX-7 to 1.50 μm, indicating that spray drying technique tends to produce sub-micro particles. The thickness of polymer layer was 10 nm–20 nm from transmission electron microscopy (TEM) images. The authors revealed that the particles were progressively coated in that the saturation of F_2602_ was not enough to make the liquid bridge crosslink, which is in accordance with the formation mechanism of particles in the spray drying process. Qiu et al. [86] conducted a similar work in terms of RDX@PVA and RDX@ carboxyfunctional terpolymer (VMCC) CSEs. They stated that the composition of microparticles could be precisely controlled by tuning the composition of explosive crystals and polymers in the precursor solution.

Lobry et al. [90] reported an innovative work on the spray flash evaporation of an oxidizer ammonium dinitramide (ADN) on two secondary explosives RDX and HMX. A liquid solution containing ADN and explosive was superheated in atomization chamber. The high temperature and pressure drop induce a sudden solvent evaporation and particle formation at the micro scale. The authors aimed to present novel insights into the formulations with an oxygen balance close to zero. Different from the raw material, ADN@HMX composite particles exhibited rod shape and ADN@RDX CSE showed needle shape, which could be attributed to the solution solvent, to a large extent. The main limiting factors are the process pressure and the solubility of the compounds in suitable solvents for spray flash evaporation process.

### 2.6. Other Fabrication Techniques

#### 2.6.1. Ultrasonic

The ultrasonic technique has a wide application in the synthesis and modification of various kinds of materials. During the ultrasound process, a rich body of cavitation bubbles is generated. These bubbles act as microreactors that grow, collapse, and create localized hot spots [35,37,75]. CSEs fabricated by this method typically feature micro-sized core explosives and nano-sized shell materials. However, the aggregation of particles after ultrasonic processing has been frequently discovered and becomes an obstacle for high coating efficiency and scaled-up production [91,92]. To overcome the problem, surface modifiers (such as PVA, Estane 3703, etc.) have been tried and proved to be essential to increase the core–shell adhesion and avoid the agglomeration of particles. Based on uniform dispersity, safe and environmentally friendly characteristics, the ultrasonic technique together with appropriate surface modification has great potential to produce compact and monodispersed core–shell composites.

Huang et al. [93] prepared TATB-coated HMX microparticles via a facile ultrasonic method. The process involved two steps, namely surface modification of HMX with the assistance of Estane and ultrasonic synthesis of HMX@TATB microcomposites. It was found that HMX and TATB were packed close through intermolecular interactions by coating. The effects of shell content, size of core particle and the amount of Estane on the morphology of the core–shell structure were studied. The contents of the shell and surface modifier can be controlled within a proper range to achieve perfect coating and decrease the energy loss. In addition, the decrease in the average particle size of core explosives is in fact detrimental to maintaining uniform coating under the constant sheath content. It was proposed that the formation of such a desirable core–shell structure could be attributed to the activated group interactions between the core–shell interfaces and the extraordinary conditions (high temperature and pressure simultaneously) conducted through high intensity ultrasound.

#### 2.6.2. Supercritical Encapsulation Method

During the past decades, the application of supercritical fluids has been an active field of research. The main motivation for this is the properties of supercritical fluids in specific pressure and temperature [23]. In particular, supercritical carbon dioxide (sc-CO_2_) is the most widely used supercritical fluid for encapsulation process due to its near-ambient supercritical temperature (*T*c = 304.2 K) for easy operation and its high solubility of most organic substances [41]. The supercritical encapsulation method stands out with two main advantages: one is the high preparation efficiency in that the separation of supercritical fluid from the product can be easily accomplished by depressurization. The other is the green and friendly production process without the use of toxic organic solvents or chemical additives. Several encapsulation processes to prepare micro-CSEs based on supercritical fluids have been developed [4,94]. He et al. [95] prepared RDX@poly(vinylidene fluoride-co-hexafluoropropylene) (VDF-HFP_22_) and RDX@polystyrene (PS) composites via a rapid expansion of supercritical solutions (RESS) method. RDX and the polymers were dissolved in sc-CO_2_ and then the dissolved material precipitated when the pressure was reduced, and the supercritical fluid expanded. RDX crystallizes first as it has lower solubility than the polymer. Subsequently, the polymer is deposited on the surface of the RDX particles. The major problem of RESS technique is that it is difficult to control the morphology of the composite particles since the formation of microparticles is extremely fast. The other challenge one should not ignore is that the utilization of polymers is relatively higher than other above-mentioned methods. The content of polymers in RDX composites is near 30%.

#### 2.6.3. Vapor Deposition Method

The vapor deposition method has been extensively adopted to prepare metastable intermixed composites (MICs). The obtained MICs (such as Al/CuO, Al/NiO and Al/Fe_2_O_3_) feature single periodicity stacked structure with intimate mixing [29,96,97,98]. Inspired by the rapid development in MIC field, the vapor deposition technique was introduced to prepare CSEs. By coating metal on the explosive surface through vapor phase deposition or magnetron sputtering methods, the explosives are free from morphology or size change, which is pivotal to integrate the multiple functionalities of core–shell materials and maintain the fundamental characteristics of core explosives. Zhou et al. [99] prepared RDX@copper oxide (CuO) core–shell particles by RF magnetron sputtering technology. CuO was expected to improve the combustion efficiency and performance of RDX-based propellants as an additive. It was found that CuO deposited onto the surface of RDX uniformly and continuously with a thickness of about 50 nm. The intimate interfacial contact between RDX and nano-sized CuO facilitates the thermal decomposition of RDX and lowers the ignition temperature. So far, the application of vapor deposition method to prepare CSEs has been confined to metal or metal oxide coating. As a result, the species of shell materials and wide application of vapor deposition method remain to be explored.

### 2.7. Comments on the Above-Mentioned Methods

Sometimes, it is difficult to choose a single method for the preparation of a certain CSE since these methods have their own advantages and drawbacks as shown in Table 2. In general, the water suspension method is a simple, versatile and straightforward physical protocol to prepare most CSEs. However, the aggregation between particles frequently occurs, especially for small-sized particles. The ultrasonic method faces the same dilemma, that the uniformity of composite particles is hard to control. Surface modifiers have been adopted to increase the dispersity of particles and improve the surface adhesion to solve the problem at some level. The crystallization coating method seems to be a practical method to decrease the agglomeration and tune the performance of CSEs. One must note that the degree of supersaturation plays such a decisive role in the process that the cooling rate should be carefully controlled. The in situ polymerization and emulsion methods are the most popular techniques currently to fabricate CSEs. These methods feature uniform and compact coating, easy scale-up, mild process conditions and profitable processing performance. A common concern with further applications is that there are limited monomers for in situ polymerization in mild conditions without destroying the structures of explosive crystals. The content of various surfactants and stabilizers should also be controlled during the emulsion process to ensure the purity and stability of the explosive composite. The other techniques, such as electrospray deposition, supercritical encapsulation, and the vapor deposition method have been recently developed for the purpose of high-performance CSEs, which are promising to prepare desirable core–shell structures with high preparation efficiency. However, the expense of these processes restricts the scale-up production to some extent. To sum up, the combination of different techniques may be an alternative to fabricate novel structured CSEs (e.g., combination of ultrasonic and in situ polymerization method, combination of self-assembly and emulsion technique). The formation mechanism of physical coating methods, including water suspension, emulsion, spray drying and ultrasonic methods, can be generally summarized into two steps: Firstly, the shell materials adhere to the surface of core explosives via hydrogen-bonding, π–π conjunction, N···O or O···O interactions under various processing conditions. Then, the shell components solidify and coat on the core surface with the removal of solvent. In the crystallization coating method, surface nucleation occurs first, and then the fine agglomerates grow on the surface of the core seed. In situ coating is a chemical polymerization process. The monomers of the shell are selectively adsorbed onto the core surface via preferential interactions, such as hydrogen bonding, π–π interaction, etc., and then polymerize in situ on the core surface under certain reaction conditions.

## 3. Compositions and Characteristics of CSEs

CSEs are core–shell structured composites with an explosive core wrapped by energetic or non-energetic shell. In general, the core explosives are high-energy and sensitive materials, such as CL-20, HMX and RDX. The shell materials are adopted to tune the sensitivity, thermal stability or mechanical properties of the core explosives. CSEs can be classified into three groups according to the types of shell material: polymer-, explosive- and novel materials-based CSEs.

### 3.1. CSEs with Polymer as Shell

Polymers have been used as binders in explosive formulations for a long time to improve the mechanical properties of the explosive charges and desensitize the high-energy explosives, composing what are known as PBXs [14,100,101]. The compatibility among the components should be paid sufficient attention to ensure the safety and performance of the composites. Polymers are expected to possess tight adhesive capability, well compatibility and reliable stability for the formulation of CSEs.

#### 3.1.1. HMX-Based CSEs

For the core components of CSEs, HMX is the most frequently used due to its high energy density, superior explosive performance and relatively low cost [8,48,102,103]. When it comes to shell components, various polymers can be considered, such as PDA, MF resin and polyurethane. By varying the polymer shell and preparation methods, the performance of corresponding CSEs could be tuned for specific applications.

**Figure 5:**
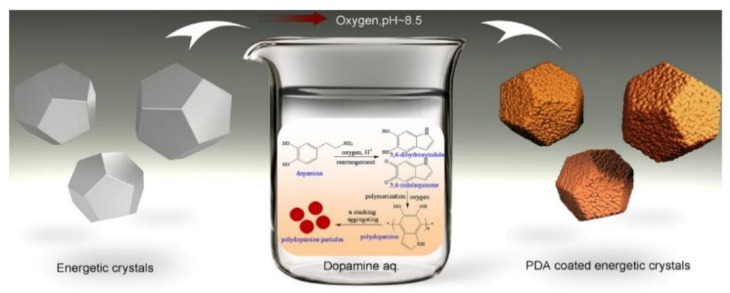
Possible deposition process of PDA on HMX crystal. Reproduced with permission from [64], copyright 2017, Elsevier.

HMX@PDA composite was the first reported CSE [64] prepared by the in situ polymerization method. The PDA shell acts as an armature to retard the phase transition of explosive crystals and enhance the mechanical strength of the composites. The authors proposed a possible formation mechanism of PDA coating on HMX surface as shown in Figure 5. Firstly, dopamine was oxidized into 5,6-dihydroxyindole and rearranged to 5,6-indolequinone under alkaline conditions. Then, the monomers concentrate on the surface of the HMX through interfacial interactions, polymerize and further assemble to PDA coating layer. It is known that the phase transition of HMX from the insensitive β-form to the sensitive δ-form is undesirable in that voids and crystal defects are induced and stability is challenged during the period [104,105]. The phase transition temperature of HMX was improved by 27.5 °C with only 0.5% PDA. The retardation of phase transition is benefit for the thermal stability of HMX composite. However, the rigid PDA shell fails to decrease the impact sensitivity at room temperature. The characterization of the degree of coverage remains a challenge for core–shell composites. Up to now, there are two methods generally accepted to evaluate the degree of coverage: the first is X-ray photoelectron spectroscopy (XPS) analysis where the surface N/C atomic ratio of the prepared CSEs, PDA and virgin explosives are compared qualitatively to confirm the effectiveness of the coating process. The other is the etching technique where a selective solvent such as acetone or ethyl acetate is adopted in the etching of the explosive cores from the CSEs. The content of explosive in the obtained solution is measured by high-performance liquid chromatography (HPLC) analysis. The amount of PDA could be determined by the formula 100%—[content of energetic crystals].

**Figure 6:**
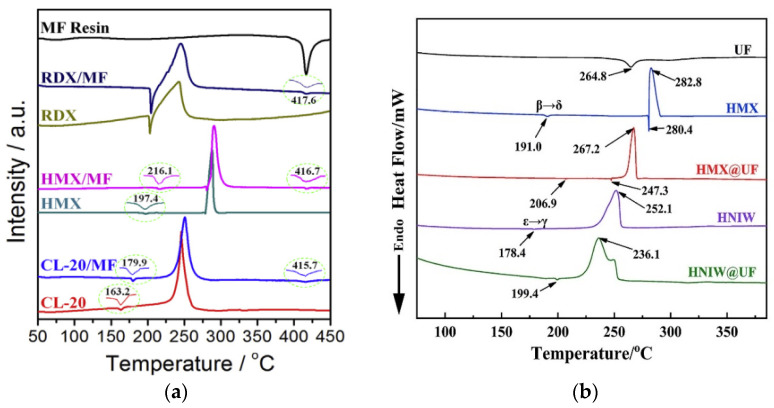
DSC curves of explosives coated by MF (**a**) and UF resin (**b**), respectively (The heating rate is 10 °C/min in (**a**,**b**). (**a**) Reproduced with permission from [60], copyright 2015, Elsevier. (**b**) Reproduced with permission from [61], copyright 2019, Elsevier.

MF, UF and MUF resins have been applied to CSEs in recent years. By in situ polymerization of resin prepolymer on the surface of HMX, RDX or CL-20, CSEs with desirable performance could be achieved. The crystal form remains unchanged during the coating process, which is quite important for the safety of polymorphic CL-20 and HMX. After coating with 3% MF resin, the phase transition as well as thermal decomposition temperature of HMX@MF increased remarkably (197.4→216.1 °C, 278.7→280.2 °C), implying improved thermal stability of the CSE composite [60]. In addition, the impact sensitivity of HMX@MF CSE (impact energy of 50% explosion probability *E*_50_ = 25.3 J) reduced visibly compared to HMX explosive (13.8 J) and physical mixtures (HMX + MF, 14.7 J). MF resin shell acts as a buffer system to dissipate the impact energy when being attacked. The high surface coating of MF resin in the CSEs produced a noticeable desensitization compared to the mixture, confirming the superiority of the core–shell structure. However, the coating of HMX with UF or MUF as shell material exhibited an unexpected reduction in thermal decomposition temperature as shown in Figure 6. HMX@PANI CSE [63] prepared via in situ polymerization and HMX@TPEE CSE (with 5/1 ratio) [76] fabricated by emulsion solvent evaporation face the similar dilemma that the peak decomposition temperature shifted lower compared with the original uncoated candidates. This may be attributed to the chemical reactivity of the core explosive with the existence of polymers at high temperatures and the possible impurities involved during the coating process. The detailed mechanism needs to be investigated further. Regarding electrostatic spark sensitivity, HMX@PANI CSEs exhibit excellent stability with *E*_50_ values twice higher than raw HMX. The fact that conductive PANI polymer could conduct static electricity to avoid aggregation on the surface of HMX may be responsible for this.

As a third example, HMX@high melting point paraffin wax (HPW)@PDA CSEs [66] possess low sensitivity and high mechanical properties with a litchi-like core@double shell structure. The composite was prepared with an inner paraffin wax shell and outer PDA shell fabricated via a facile water suspension and in situ polymerization method sequentially. The authors provided a unique perspective on the intermolecular interactions between HMX and the polymer binder. The contact angle slightly increased with HPW coating (from 66.63° for HMX/copolymer of chlorotrifluoroethylene and vinylidene fluoride (F_2314_) to 70.55° for HMX@HPW/F_2314_), indicating that the coating with paraffin wax resulted in a decrease in interfacial interaction. The PDA coating improves the compatibility between HMX@HPW@PDA and F_2314_, attributing to the hydrogen bonding with –OH groups in PDA molecule as proton donors and –F groups in fluoropolymer chains as proton acceptors. Based on a parameter introduced by Kubát [106] in terms of the interface energy loss, the calculation results showed that the flow of HPW filled the voids between the explosive and the binder, and a higher molding temperature increased the interfacial interaction of explosives and F_2314_.

**Figure 7:**
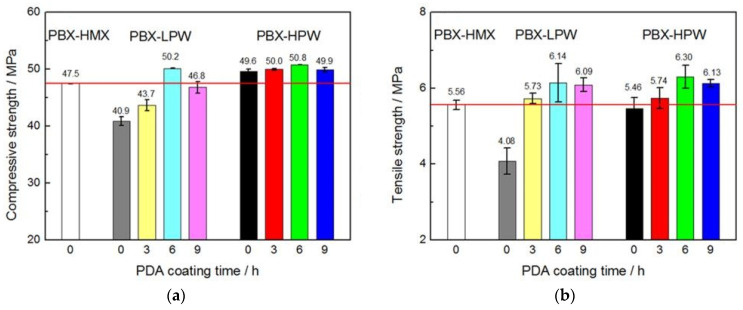
Compressive- (**a**) and tensile strength (**b**) of HMX, HMX@wax and HMX@wax@PDA-based energetic composites. Reproduced with permission from [66], copyright 2020, American Chemical Society.

In terms of thermal properties, the polymorphic phase transition temperature (*T*_0_) of HMX@HPW CSE slightly increased by 1.1 °C, and the further coating with PDA-6 h provided an evident retardation of phase transition temperature by 12.2 °C. The mechanism of enhanced phase transition temperature may be that the strong interface interactions blocked the formation of δ-nuclei at the crystal surface. However, the coatings had little effect on the thermal decomposition temperature of HMX@HPW@PDA composites. Compared to HMX virgin explosive, the HMX@HPW@PDA particles demonstrated a 160% increase in impact energy *E*_BAM_ from 5 J to 13 J and a significant decrease in friction sensitivity from 92% to 20%, implying that the surface coating with core–shell structure is very favorable for the safety performance of explosives. As for mechanical properties, the litchi-like HMX@HPW@PDA CSEs also exhibited far superior mechanical strength than those of the corresponding HMX@HPW and the raw HMX explosive. The influence of the melting point of paraffin wax and PDA coating time on the mechanical strength were explored. As shown in Figure 7, the relatively high melting point of paraffin wax was in favor of compressive and tensile strength as the paraffin wax with high plasticity could fill the voids. With PDA coating for 3 h, the compressive and tensile strengths increased, compared with the corresponding PBX-low melting point paraffin wax (LPW) and PBX-HPW. The composites with PDA coating time of 6 h exhibited improvements in compressive strength and tensile strength of 6.9% and 13.31%, respectively. With the further increase in PDA coating time to 9 h, the compressive and tensile strengths slightly decreased. The preparation of the HMX-based core@double shell composite proved the superiority of core–shell structure that a synergistic effect of the remarkable desensitization of the paraffin wax and the strong interfacial adhesion of PDA could be achieved simultaneously.

#### 3.1.2. TATB-Based CSEs

TATB is a moderately powerful, thermally stable and insensitive explosive [15,107,108,109]. TATB crystals consist of graphite-like sheets with considerable intermolecular interactions between layers [34,58]. However, TATB crystals suffer from large deformation when exposed to the thermal physical environment due to their unique structure, which restricts long-term storage and transport. PDA was adopted as shell material to prepare TATB-based CSE via the in situ polymerization method. Lin et al. [52] conducted a systematic study on the mechanical properties such as the storage modulus, creep resistance and compression behavior, of TATB and TATB-based CSEs. The compressive and Brazilian tests revealed improved compressive strength (48–61% increase), compressive fracture energy (79–105% increase), tensile strength (39–73% increase), and tensile fracture energy (100–219% increase) for the TATB@PDA composites, compared with the virgin explosives. In addition, plenty of functional groups including amino, hydroxyl and catechol groups, were integrated at the PDA coating surface, which could behave as heterogeneous nucleation centers for adhesion of binders [110].

**Figure 8:**
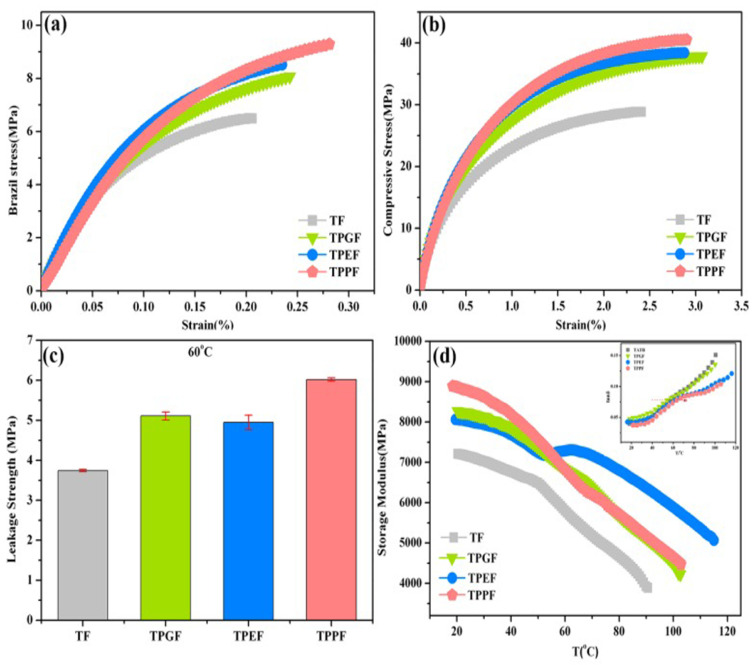
Stress–strain curves of (**a**) Brazil test, (**b**) compression test, (**c**) leakage strength, and (**d**) storage modulus for TATB-based polymer-bonded explosive (PBX) with and without grafting. (TF: TATB/F_2314_, TPGF: TATB@PDA@GAP/F_2314_, TPEF: TATB@PDA@PEG/F_2314_, TPPF: TATB@PDA@PTMEG/F_2314_). Reproduced under the terms of the CC-BY Creative Commons Attribution 4.0 International Licence (https://creativecommons/licenses/by/4.0, accessed on 19 August 2021). Reproduced with permission from [58], copyright 2019, MDPI.

**Figure 9:**
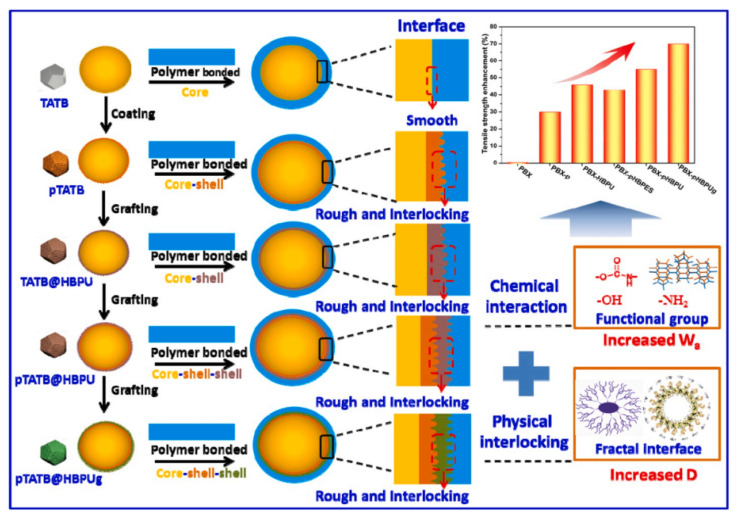
Schematic representation of the interfacial interactions in TATB-based PBX composites. Reproduced with permission from [55], copyright 2020, Elsevier.

A further study was reported by taking the PDA-modified surface as a secondary reaction platform for the grafting of three polymer binders: GAP, PEG, and PTMEG [58]. The composites demonstrated superior mechanical performance over virgin TATB, especially for the PTMEG-grafting CSE (Figure 8). The Brazil strength of PTMEG-grafted PBX increased by 40.9%, and the compressive strength increased by 40.1% as compared with TATB-based PBX. In terms of thermal stability, the grafting of the polymers induced a slight lower shift of peak decomposition temperature ranging from 3.7 to 4.6 °C, which may be attributed to the lower relative decomposition temperature of the polymers compared with TATB. He et al. [55] conducted similar work by in situ grafting of HBPs on a polydopamine (PDA) surface via the “grafting from” strategy to fabricate TATB. A similar mechanical enhancement was observed. Compared with the pTATB (TATB@PDA) structure, the grafting of hyperbranched polyurethane (HBPU) on PDA shell leads to a stronger interfacial interaction and more robust adhesion capability with fluoropolymer than the neat PDA shell. A physical “interlocking block” model formed by the rough and fractal interface was proposed, and is displayed in Figure 9. When HBPU was attached to TATB surface bridged through PDA film, more anchor points were created in the rough and fractal interface surfaces, which further played a coordinated role in enhancing the interfacial interaction. In summary, the preparation of core–shell structure by bio-inspired PDA material and further polymer grafting provides an efficient route for the interfacial and mechanical enhancement of TATB.

#### 3.1.3. CSEs Based on Other Explosives

**Figure 10:**
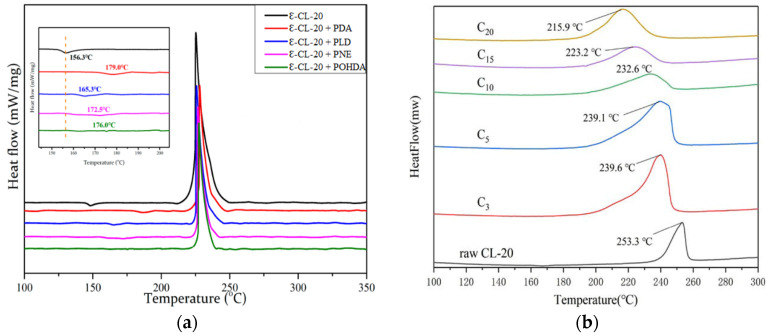
DSC curves for raw CL-20 and different composites (samples C_3_, C_5_, C_10_, C_15_, and C_20_ in **b** denote that the weight ratios of CAB in the composites are 3%, 5%, 10%, 15% and 20%; the heating rate in (**a**,**b**) are 5 °C/min and 10 °C/min, respectively). (**a**,**b**) Reproduced under the terms of the CC-BY Creative Commons Attribution 4.0 International Licence (https://creativecommons/licenses/by/4.0, accessed on 19 August 2021). (**a**) Reproduced with permission [57]. Copyright 2020, MDPI. (**b**) Reproduced with permission [78]. Copyright 2020, American Institute of Physics.

CL-20 is one of the explosive elements with the highest energy level so far [111]. CL-20 possesses four crystal forms: ε, α, β and γ form at ambient temperature, and the energy density of ε-CL-20 is the highest with the lowest sensitivity [112,113,114]. The phase transition of CL-20 (ε→γ) under thermal stimulation leads to an expansion in volume, which may stimulate hot spots and lead to explosive deflagration [115,116]. Inspired by the strong chemical adhesion of mussels, PDA was adopted to construct CL-20-based CSEs [57]. As shown in Figure 10a, the PDA coating has a remarkable improvement effect on the phase transition temperature of CL-20. The transformation of CL-20 crystal was retarded by 22.7 °C, denoting an enhanced thermal stability of CL-20 composite. Meanwhile, the friction sensitivity of the CSE composite decreased from 96% to 48% compared with raw CL-20. However, the coating did nothing for the impact sensitivity, which was attributed to the rigid PDA shell and the irregular crystal shape and defects. Wang et al. [78] reported a novel CL-20@CAB CSE by premix membrane emulsification method. CAB possesses good leveling and film-forming properties, causing a successful thin film deposition on the surface of CL-20. It can be observed from Figure 10b that the decomposition temperature of the composite decreases gradually with increasing weight ratios of CAB, indicating that the reaction activity increases, and the reaction rate accelerates. One can find that the impact sensitivity decreases with the increasing content of CAB. The fact that the particle size reduces as the CAB content increases may be responsible for this.

RDX is another attractive secondary explosive applied to CSEs due to its good energetic performance and reasonable cost [87,95,102,117]. A representative RDX@PMMA CSE [118] was prepared by Jia et al. via water suspension and emulsion polymerization method. Both of the methods provided a successful coating of PMMA on the surface of RDX, and the composite produced by the emulsion polymerization method possessed a more uniform size distribution and a narrower grain size. With the addition of the PMMA shell, the peak decomposition temperature showed a slight increase for the composites. In terms of impact sensitivity, the drop height (*H*_50_) values of RDX/PMMA particles prepared by the two methods increased by 8.6 cm and 15.4 cm, respectively. The desensitization effect of emulsion polymerization is more significant in that the uniform dispersed particles reduce the stress concentration between the particles and efficiently prevent the formation of local hot spots. Spray drying method was used to fabricate RDX@polyvinyl acetate (PVAc) and RDX@vinyl resin (VMCC) CSEs [86]. The crystallization of the small RDX crystals and formulation are achieved simultaneously during the process. SEM images show that most of the particles are below 1 μm as compared with the virgin RDX particles (5–30 μm). Both of the RDX-based composites feature reduced shock sensitivity, which may be attributed to the small crystal size as well as small void size (~250 nm).

### 3.2. CSEs with Explosive as Shell

High energy density is always a primary goal for explosives. To reduce the energy loss as much as possible during the coating process, there is an elegant method to tune the performance of explosives by constructing core–shell structure with insensitive explosives as the shell material. TNT, NTO, and TATB are used to coat various sensitive explosives, such as CL-20, HMX and RDX. Given the weak intermolecular interaction among explosives, the key challenge is to prepare core–shell composites with high surface coverage and strong coating strength with an appropriate fabrication process.

HMX@NTO composite is a typical explosive@explosive CSE fabricated by crystallization coating in alcohol or water-*N*-methyl-2-pyrrolidone (NMP) solvent [82]. The growth rate of the HMX coating increased with rising concentration of NTO, but then began to decrease due to high agglomeration. It was found that high supersaturation was in favor of uniform particle deposition on the surface of HMX. The impact sensitivity of HMX@NTO CSE was 8.2 J with a coating thickness of 3 μm, superior to that of HMX (4.6 J), indicating that the safety has been improved observably for HMX-based composite. To improve the safety of RDX, an energetic polymer (HP-1) together with TNT were introduced to coat RDX by combining the solvent–nonsolvent and water suspension methods [50]. HP-1 reduced the surface tension of coating materials and improved the adhesive ability on the surface of RDX. After coating with 2.5 wt% TNT and 0.5 wt% HP-1, the drop height (*H*_50_) was increased by 57%, and the friction probability reduced by 54%. Meanwhile, the thermal stability of the coated composites improved slightly. Remarkably, the influence of coating on the energy performance of RDX is negligible because its estimated explosion heat is only reduced by 0.93%.

HMX@TATB CSE is another representation worthy of mention. So far, there are four approaches reported to coat sensitive high explosive with the TATB shell, including in situ coating, water suspension, ultrasonic, and spray drying approaches. In situ coating of HMX crystals with TATB was carried out by amination of 1,3,5-trichloro-2,4,6-trinitrobenzene (TCTNB) with dry ammonia gas [119]. The impact sensitivity and friction sensitivity of HMX@TATB CSE with 10% shell content were 24% and 0%, respectively. The thermal decomposition peak temperature of HMX@TATB CSE (285.6 °C) was higher than the physical mixed sample (283.3 °C), indicating that the compact core–shell structure contributed to high thermal stability. The fact that the formation of core–shell structure induces a modest cage effect may be responsible for the improved thermal stability after coating. However, the coating has little effect on the thermal decomposition performance of HMX, which may be caused by the impurities during the process and a relatively low utilization of raw materials. A series of studies [52,58,68,88,92,93] reveal that the particle size of TATB plays a key role in the effectiveness of coating. Sub micro-TATB was utilized to coat CL-20 via a water suspension process [51]. Due to the cushioning and lubricating effects of TATB shell, the *H*_50_ value of the composite increased to 49.6 cm, more than three times higher than the 16 cm of the original CL-20, implying that the desensitization strategy by core–shell coating is effective. Ultrasonic approach was utilized to prepare HMX@nano-TATB microparticles [93]. The impact and friction sensitivity of HMX@TATB composites with 10% shell content are 75 cm and 8%, respectively, evidently better than those of HMX and the physical mixture. It can be concluded that ultrasonic method creates an effective core–shell structure, hence the CSEs are less sensitive to mechanical stimuli. In addition, the thermal decomposition temperature became lower with the increase in shell content. This result implies that the evolution of coverage degree of core–shell structure is accompanied by an opposite trend in decomposition temperature. Facile, continuous, and large-scale production of core–shell HMX@TATB composites was achieved by a spray drying process [88]. The utilization of TATB shell was evidently decreased from the previously reported 15 wt% to 8 wt%, thus ensuring the energetic performance of the explosive. More importantly, the impact and friction sensitivity results showed that the prominent stability of these core–shell microparticles with low shell content can be maintained.

**Figure 11:**
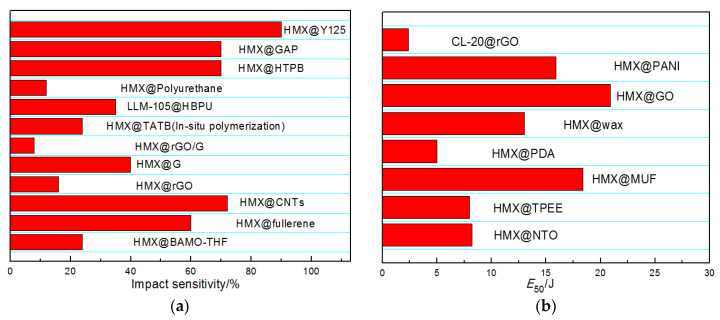

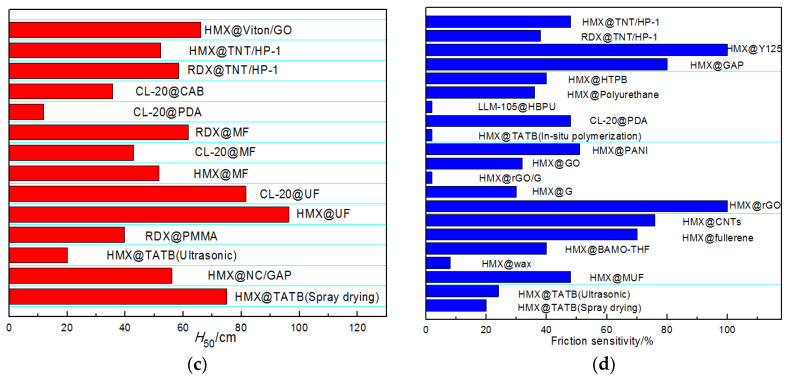
Impact sensitivity and friction sensitivity of some typical CSEs: (**a**) Impact sensitivity denoted by explosion probability according to GJB-772A-97 standard method; (**b**) Impact sensitivity denoted by *E*_50_ according to the Bundesanstalt für Materialprüfung (BAM) method [100]; (**c**) Impact sensitivity denoted by *H*_50_ according to GJB-772A-97 standard method; (**d**) Friction sensitivity.

As shown in Figure 11, there are three methods of characterizing impact sensitivity. Special attention must be paid to performance comparisons of certain CSEs since the performance of the composite is determined by the particle size, morphology, the shell content and preparation method. The differences may influence the thermal stability and sensitivity of the composites significantly, and therefore it is difficult to conduct a more intuitive comparison among different CSE systems. Despite that, the data do provide certain basic characteristics of CSEs and the inherent correlations between different CSE systems. Table 3 summarizes some important CSEs in terms of their preparation method and property improvements. It is obvious that rGO and MF coatings could achieve outstanding desensitization effect with minimal shell content among the listed CSEs under the same experimental conditions.

### 3.3. CSEs with Novel Materials as Shell

Apart from polymers and conventional explosives, some novel materials, such as CuO, graphene (G), graphene oxide (GO) and carbon nanotubes (CNTs) were introduced to prepare CSEs due to their unique electrical, thermal, mechanical and structural properties [70,99,103,120,121]. rGO together with G were selected as shell materials to coat HMX through an in situ chemical reduction coating method [43]. The differential thermal analysis (DTA) results showed that the thermal peaks of HMX changed little, indicating that the added G and rGO were compatible with HMX. The impact sensitivity of raw HMX decreased from 100% to 8% and the friction sensitivity reduced from 100% to 0% with the addition of 1.0 wt% GO and 1.0 wt% G, implying that rGO sheets along with graphene are promising to be utilized as co-desensitizers for nitramine explosives. Subsequently, the research group conducted a study in terms of various explosive systems to tune their thermal stability and sensitivity, including insensitive HMX@G composites [121], HMX@GO composites [103], CL-20@rGO composites [39] and HMX@Viton/GO composites [48]. All the results indicate that these carbon materials can be utilized to desensitize the explosives significntly with a low additive amount.

Many kinds of nanomaterials have been developed and tested for catalytic decomposition of explosives, and thereby improve the combustion efficiency and performance of propellants. CuO was deposited onto the surface of superfine RDX particles to form RDX@CuO CSE through RF magnetron sputtering technology [99]. It was found that intimate interfacial contact was realized with a thin film of 50 nm, and morphology or size change of RDX was avoided. CuO can catalyze the thermal decomposition of RDX and lead to decreased decomposition temperature by 24.8 °C.

**Figure 12:**
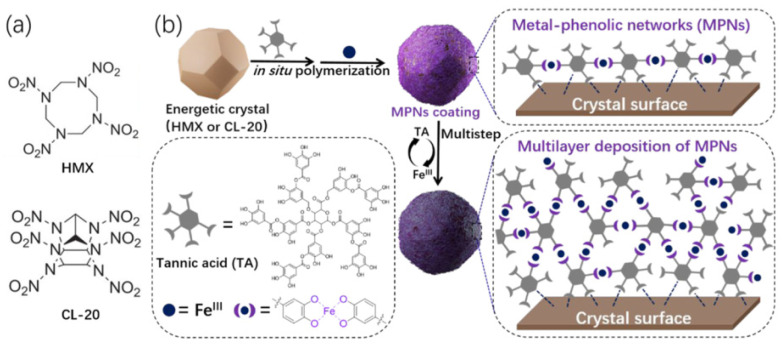
(**a**) Molecular structures of the typical energetic molecules HMX and CL-20; (**b**) Schematic representation of the preparation of the surface-modified energetic crystal via multilayer deposition of MPNs. Reproduced with permission from [65], copyright 2020, Elsevier.

Metal-phenolic networks (MPNs) are an emerging class of supramolecular coatings formed through coordination chemistry, which have strong adhesive attachment to diverse organic surfaces [44]. Tannic acid (TA)-based MPNs were utilized to coat HMX via in situ noncovalent decoration of polyphenols and Fe^III^ as shown in Figure 12 [65]. HMX surface was regularly coated through layer-by-layer deposition and the thickness of coating could be controlled by tuning the coating cycles. The phase transition (β→δ) temperature of HMX was significantly improved by 42.3 °C with a low shell content of 1.8 wt%. This surface modification strategy features high efficiency and mild preparation conditions, which provide the potential for large-scale fabrication of high explosives.

### 3.4. Challenges and Prospects

As mentioned above, a variety of polymers, insensitive explosives and some novel materials have been developed as suitable shell materials for CSEs. Thermal stability, sensitivity and mechanical properties are the greatest concerns for CSEs. The preparation of core–shell structures can imporove the comprehensive performance of the explosives significantly. For polymorphic explosive crystals like CL-20 and HMX, the inclusion of coating materials, such as PDA, MF resin and TATB, contributes to the retardation of phase transition and thereby improves the thermal stability of the explosives. As shown in Table 3, most CSEs with polymers or heat-resistant insensitive explosives as the shell could achieve pronounced desensitization with no more than 5% shell content while preserving the energy output of explosives. It should be noted that PBXs based on some of the CSE particles display improved roughness, storage modulus, as well as creep resistance due to the strong interfacial adhesion. TATB@PDA CSE is a very typical example. 

However, several challenges remain to be resolved. For example, how best can the performance of these micro-CSEs, including power, stability, and mechanical properties be integrated? Incorporating the merits of different methods may provide opportunities to solve the contradictions. The ultrasonic technique is an efficient method for explosive surface pre-treatment, and preparation of desirable core–shell structures together with other methods, such as in situ polymerization, emulsion and spray drying. In addition, the performance studies of CSEs mainly focus on thermal stability and sensitivity, whereas the mechanical strength and detonation performance data are still lacking. The limitations include the great quantity of testing samples required, special equipment required, and the safety concerns of explosion and mechanical strength tests. High precision calculation methods as well as experimental measurements of detonation performance are urgently needed. The relationships between the microstructure of CSEs and their detonation performance could be explored further in future studies. The mechanical properties tests for explosives are based on PBXs formula currently, thus the standard component percentage and measurement method need to be clarified. To clearly solve these questions, there is still much work to be done.

## 4. Conclusions

The core–shell strategy gives an alternative approach to solve the contradiction of energy and safety for explosives. The most attractive advantage of CSEs is that high surface coverage and strong coating strength can be achieved with minimized content of shell materials. Owing to this merit, thermal stability, mechanical strength and insensitivity of explosives can be improved remarkably, and the energy output can be maintained. Close contact is the essential difference between CSEs and physical mixing composites. In the past decade, many enticing CSEs have been discovered, and so far, the preparation and characterization procedures for CSEs are slowly being established. The preparation methods were summarized herein and the advantages and disadvantages of different techniques were revealed. In addition, the most typical examples of CSEs in terms of their compositions and characteristics were introduced. The preparation method greatly depends on the components of CSEs in view of the compatibility of the components and the requirements of CSEs, and a mild or solution preparation method is preferred generally.

Even though much has been achieved regarding the preparation and properties of CSEs, there are still some fundamental issues waiting for solutions. The balance of high energy, low sensitivity and good mechanical strength remains a challenge for CSEs, and other shell materials should be explored. The formation mechanism of many types of CSEs prepared by different methods needs to be clarified. Further studies are expected to simulate and calculate the interfacial interactions between core and shell materials, to analyze the formation mechanism and further guide the design of the formulation of CSEs. The influence of the micro-structure of CSEs on their mechanical strength and detonation performance should be further explored systematically. Additionally, the inclusion of additives, such as the surface surfactant, stabilizer and emulsifier, during the coating process brings a potential risk for the shell materials to be integrated with the additives. Special attention should be paid to the impurities and their effects on the performance of the composites. The future of this field still poses many challenges, but there is no denying that the core–shell strategy will certainly play a very important role in assuring the development of explosives, pyrotechnics, and propellants.

## Figures and Tables

**Table 2:** Characteristics of the products prepared by water suspension, emulsion, spray drying and other methods.

Product	Preparation Methods	Size of Core [Diameter, μm]	Feature	Comments	Contributor
RDX@TNT/HP-1	Water suspension	70	A coarse and continuous film coating over RDX surface.	Rough surface and nice coating structure could be achieved. Sometimes aggregations exist.	[50]
LLM-105@fluoropolymer	Water suspension	60	Spherical morphology, rough surface and few agglomerates.		[27]
HMX@TPEE	Emulsion solvent evaporation	25	Compact and coherent spherical particles with many tiny holes.	The use of emulsifiers has significant influence on the morphology of microspheres.	[76]
CL-20@CAB	Premix membrane emulsification	78	Dumbbell-shaped composites with two balls sticking together.		[78]
HMX@TATB	Spray drying	10–25	The surface of core–shell composites presented a coarse and continuous morphology.	A highly efficient one-step process to produce core–shell micro-particles.	[88]
FOX-7@F_2602_	Spray drying	20–69	The particle size decreased significantly after coating with the thickness of shell layer about 10–20 nm.		[89]
HMX@NTO	Crystallization coating	200–300	NTO crystallized onto the surface of HMX as the nucleation center homogeneously.	The specific crystal morphology and narrow crystal size distribution can be achieved.	[80]
HMX@TATB	Ultrasonic	90–120	Rough surface and homogeneous coating.	A mild and suitable process to prepare micro-CSEs. Dispersant is commonly used to avoid aggregation.	[93]
RDX@VDF-HFP_22_	Supercritical encapsulation		Smooth and homogeneous thin film was obtained.	Green production process with high preparation efficiency but few aggregates.	[95]
RDX@CuO	RF magnetron sputtering		CuO covered the RDX particle intimately and uniformly.		[99]

**Table 3:** Preparation methods and properties improvement of some CSEs.

Product	Preparation Methods	Shell Content/%	Increment of Phase Transition Temperature/°C	Increment of Peak Decomposition Temperature/°C	Improvement Percentage of *H*_50_/%	Improvement Percentage of *E*_50_/%	Contributor
HMX@PDA	In situ polymerization	0.5	26	0.2	0		[64]
HMX@MF	In situ polymerization	2.9	18.7	3.2	83		[60]
HMX@UF	In situ polymerization	4.3	15.9	−15.6	246		[61]
HMX@MUF	In situ polymerization	5.0	NA	NA	240		[62]
HMX@PANI	In situ polymerization	3.1	17.2	−2.4		189	[63]
HMX@TPEE	Emulsion solvent evaporation	5.0	NA	−1.4		57	[76]
HMX@HPW@PDA	Water suspension and in situ polymerization	2.0	11.3	0		117	[66]
RDX@MF	In situ polymerization	3.0		2.7	85		[60]
RDX@PVAc	Spray drying	17.0		NA		60 (Shock sensitivity)	[86]
RDX@VMCC	Spray drying	17.0		NA		32 (Shock sensitivity)	[86]
RDX@PMMA	Water suspension	3.0		0.37	35		[118]
RDX@PMMA	Emulsion polymerization	3.0		2.38	63		[118]
CL-20@MF	In situ polymerization	3.0	16.7	6.1	163		[60]
CL-20@PDA	In situ polymerization	1.6	22.7	NA	0		[57]
CL-20@UF	In situ polymerization	3.9	NA	−16	350		[61]
CL-20@CAB	Premix membrane emulsification	3.0	NA	−13.7	102		[78]
HMX@NTO	Crystallization coating	6.0	NA	NA	78		[80]
RDX@TNT/HP-1	Water suspension	2.5/0.5		0.6	57		[50]
HMX@TATB	Ultrasonic	15	NA	NA	>348		[93]
HMX@TATB	Spray drying	8.0	NA	NA	>239		[88]
HMX@TATB	In situ coating	10.0	NA	NA	75		[119]
CL-20@TATB	Water suspension	5.0	NA	NA	210		[51]
		35.0	10.4	0.5	272		
CL-20@rGO	In situ reduction	2.0	18.48	0.5	171		[39]
HMX@Viton	Water suspension	5.0	NA	0.8	143		[48]
HMX@Viton/GO	Water suspension	4/1	NA	−0.2	237		[48]
HMX@rGO/G	In situ reduction	2.0	NA	−0.2	92		[43]
HMX@MPNs	In situ polymerization	1.8	42.3	NA			[65]
RDX@CuO	Vapor deposition	54		−24.8			[99]

## References

[1] Elbeih A., Zeman S., Jungová M., Vávra P. (2013). Attractive nitramines and related PBXs. Propellants Explos. Pyrotech..

[2] Sikder A.K., Sikder N. (2004). A review of advanced high performance, insensitive and thermally stable energetic materials emerging for military and space applications. J. Hazard. Mater..

[3] Duan B.H., Liu N., Wang B.Z., Lu X.M., Mo H.C. (2019). Comparative theoretical studies on a series of novel energetic salts composed of 4,8-dihydrodifurazano[3,4-b,e]pyrazine-based anions and ammonium-based cations. Molecules.

[4] Yao Y.Y., Lin Q.H., Zhou X.L., Lu M. (2021). Recent research on the synthesis pentazolate anion cyclo-N_5_^−^. FirePhysChem.

[5] Zhao X., Li Z., Zhang J., Gong F., Huang B., Zhang Q., Yan Q.L., Yang Z. (2021). Regulating safety and energy release of energetic materials by manipulation of molybdenum disulfide phase. Chem. Eng. J..

[6] Hoffman D.M., Caley L.E. (1986). Polymer blends as high explosive binders. Polym. Eng. Sci..

[7] Manning T.G., Strauss B. (2003). Reduction of Energetic Filler Sensitivity in Propellants through Coating. U.S. Patent.

[8] Antoine E.D.M., Richard H.B. (2004). Crystallization and characterization of RDX, HMX, and CL-20. Cryst. Growth Des..

[9] Hunt E.M., Jackson M. Coating and characterization of energetic materials. Proceedings of the ASME 2010 International Mechanical Engineering Congress and Exposition.

[10] Zhang J., Shreeve J.M. (2016). Time for pairing: Cocrystals as advanced energetic materials. CrystEngComm.

[11] Misasi J.M., Jin Q., Knauer K.M., Morgan S.E., Wiggins J.S. (2017). Hybrid POSS-hyperbranched polymer additives for simultaneous reinforcement and toughness improvements in epoxy networks. Polymer.

[12] He G., Li X., Jiang Y., Dai Y., Xu R., Zeng C., Tu X., Yang Z. (2020). Bioinspired hierarchical interface design for improved mechanical and safety properties in energetic polymer composites. J. Mater. Sci..

[13] Liu Z.W., Xie H.M., Li K.X., Chen P.W., Huang F.L. (2009). Fracture behavior of PBX simulation subject to combined thermal and mechanical loads. Polym. Test..

[14] Yang G.S., Zhou Z.J., Zhang X.Y., Pan J.H., Liu L.P. (2016). Polymer bonded explosives (PBXs) with reduced thermal stress and sensitivity by thermal conductivity enhancement with graphene nanoplatelets. Compos. Sci. Technol..

[15] Lin C., Tian Q., Chen K., He G., Zhang J., Liu S., Almasy L. (2017). Polymer bonded explosives with highly tunable creep resistance based on segmented polyurethane copolymers with different hard segment contents. Compos. Sci. Technol..

[16] Liu Y., Peng L., Su Z. (2007). Core-shell attapulgite@polyaniline composite particles via in situ oxidative polymerization. Synth. Met..

[17] Ma X., Li Y., Hussain I., Shen R., Yang G., Zhang K. (2020). Core-shell structured nanoenergetic materials: Preparation and fundamental properties. Adv. Mater..

[18] Huang B., Xue Z., Fu X., Yan Q.L. (2021). Advanced crystalline energetic materials modified by coating/intercalation techniques. Chem. Eng. J..

[19] Zuo C., Wang L., Tong Y., Shi L., Ding W., Li W. (2021). Co-deposition of pyrogallol/polyethyleneimine on polymer membranes for highly efficient treatment of oil-in-water emulsion. Sep. Purif. Technol..

[20] Jiang J., Zhu L., Zhu L., Zhu B., Xu Y. (2011). Surface characteristics of a self-polymerized dopamine coating deposited on hydrophobic polymer films. Langmuir ACS J. Surf. Colloids.

[21] Gong Q.H., Gao T.T., Hu T.T., Zhou G.W. (2019). Synthesis and electrochemical energy storage applications of micro/nanostructured spherical materials. Nanomaterials.

[22] Zhang J., Du Y., Dong K., Su H., Zhang S., Li S., Pang S. (2016). Taming dinitramide anions within an energetic metal–organic framework: A new strategy for synthesis and tunable properties of high energy materials. Chem. Mater..

[23] Xiao Z., He W., Ying S., Zhou W., Xu F. (2014). Current trends in energetic thermoplastic elastomers as binders in high energy insensitive propellants in China. Sci. Technol. Energ. Mater..

[24] Liu W., Wang Y., Wang P., Li Y., Jiang Q., Hu X., Wei Y., Qiu Y., Shahabadi S., Lu X. (2017). A biomimetic approach to improve the dispersibility, interfacial interactions and toughening effects of carbon nanofibers in epoxy composites. Compos. Part B Eng..

[25] Chen S., Zhang J., Zhou J., Zhang D., Zhang A. (2018). Dramatic toughness enhancement of benzoxazine/epoxy thermosets with a novel hyperbranched polymeric ionic liquid. Chem. Eng. J..

[26] He G., Li X., Dai Y., Yang Z., Zeng C., Lin C., He S. (2019). Constructing bioinspired hierarchical structure in polymer based energetic composites with superior thermal conductivity. Compos. Part B Eng..

[27] Yang Z., Lin C., Gong F., Zeng C., Zhang J., Huang F. (2019). Effects of crystal quality and morphology on the mechanical performance of LLM-105 based PBXs. Propellants Explos. Pyrotech..

[28] Zhang J., Hwang J., Antonietti M., Schmidt B. (2019). Water-in-water pickering emulsion stabilized by polydopamine particles and cross-linking. Biomacromolecules.

[29] He W., Li Z.H., Chen S., Yang G., Yang Z., Liu P.J., Yan Q.L. (2020). Energetic metastable n-Al@PVDF/EMOF composite nanofibers with improved combustion performances. Chem. Eng. J..

[30] Gong Q.H., Wang H.Q., Song W.H., Sun B., Cao P., Gu S.N., Sun X.F., Zhou G.W. (2021). Tunable synthesis of hierarchical yolk/double-shelled SiOx@TiO_2_@C nanospheres for high-performance lithium-ion batteries. Chem.-Eur. J..

[31] Zhang P., Hu W., Wu M., Gong L., Tang A., Xiang L., Zhu B., Zhu L., Zeng H. (2019). Cost-effective strategy for surface modification via complexation of disassembled polydopamine with Fe(III) ions. Langmuir ACS J. Surf. Colloids.

[32] Chen L., Liu J., He W. (2021). Bio-inspired fabrication of energetic crystals@cellulose nanofibers core-shell composites with improved stability and reduced sensitivity. Compos. Commun..

[33] He G., Yang Z., Pan L., Zhang J., Liu S., Yan Q.L. (2017). Bioinspired interfacial reinforcement of polymer-based energetic composites with a high loading of solid explosive crystals. J. Mater. Chem. A.

[34] Liu J.H., Yang Z.J., Liu S.J., Zhang J.H., Liu Y.G. (2018). Effects of fluoropolymer binders on the mechanical properties of TATB-based PBX. Propellants Explos. Pyrotech..

[35] Lin C., Huang B., Gong F., Yang Z., Liu J., Zhang J., Zeng C., Li Y., Li J., Guo S. (2019). Core@double-shell structured energetic composites with reduced sensitivity and enhanced mechanical properties. ACS Appl. Mater. Interfaces.

[36] An C.W., Guo X.D., Song X.L., Wang Y., Li F.S. (2009). Preparation and safety of well-dispersed RDX particles coated with cured HTPB. J. Energ. Mater..

[37] Wang Z., Guo X., Wu F., Yan T. (2016). Preparation of HMX/TATB composite particles using a mechanochemical approach. Propellants Explos. Pyrotech..

[38] Akiki M., Menon S. (2015). A model for hot spot formation in shocked energetic materials. Combust. Flame.

[39] Huang B., Xue Z., Chen S., Chen J., Li X., Xu K., Yan Q.L. (2020). Stabilization of ε-CL-20 crystals by a minor interfacial doping of polydopamine-coated graphene oxide. Appl. Surf. Sci..

[40] Li H., Luo Y., Yu F., Zhang H. (2021). In-situ construction of MOFs-based superhydrophobic/superoleophilic coating on filter paper with self-cleaning and antibacterial activity for efficient oil/water separation. Colloids Surf. A Physicochem. Eng. Asp..

[41] Mishima K., Matsuyama K., Tanabe D., Yamauchi S., Young T.J., Johnston K.P. (2000). Microencapsulation of proteins by rapid expansion of supercritical solution with a nonsolvent. AIChE J..

[42] Zhu W., Ren J., Wang Z., Han D., Yang H., Guo X. (2015). Synthesis of “brain-like” hierarchical porous microspheres by emulsion-solvent evaporation. Mater. Lett..

[43] Niu C., Jin B., Peng R., Shang Y., Liu Q. (2017). Preparation and characterization of insensitive HMX/rGO/G composites via in situ reduction of graphene oxide. RSC Adv..

[44] Rahim M.A., Kristufek S.L., Pan S., Richardson J.J., Caruso F. (2019). Phenolic building blocks for the assembly of functional materials. Angew. Chem. Int. Ed..

[45] Yu Q., Zhao C., Zhu Q., Sui H., Yin Y., Li J. (2020). Influence of polydopamine coating on the thermal stability of 2,6-diamino-3,5-dinitropyrazine-1-oxide explosive under different heating conditions. Thermochim. Acta.

[46] Chen J., Wang J.Y., Wang B.G., Huang H. (2009). Study on preparation process of ε-HNIW booster explosive by water slurry method. Chin. J. Explos. Propellants.

[47] An C., Wang J., Xu W., Li F. (2010). Preparation and properties of HMX coated with a composite of TNT/energetic material. Propellants Explos. Pyrotech..

[48] Wang J., Ye B., An C., Wu B., Li H., Wei Y. (2016). Preparation and properties of surface-coated HMX with viton and graphene oxide. J. Energ. Mater..

[49] Kasprzyk D.J., Bell D.A., Flesner R.L., Larson S.A. (2015). Characterization of a slurry process used to make a plastic-bonded explosive. Propellants Explos. Pyrotech..

[50] An C.W., Li F.S., Song X.L., Wang Y., Guo X.D. (2009). Surface coating of RDX with a composite of TNT and an energetic-polymer and its safety investigation. Propellants Explos. Pyrotech..

[51] Yang Z., Li J., Huang B., Liu S., Huang Z., Nie F. (2014). Preparation and properties study of core-shell CL-20/TATB composites. Propellants Explos. Pyrotech..

[52] Lin C., Gong F., Yang Z., Pan L., Liu S., Li J., Guo S. (2018). Bio-inspired fabrication of core@shell structured TATB/polydopamine microparticles via in situ polymerization with tunable mechanical properties. Polym. Test..

[53] Gong Q.H., Li Y.J., Huang H., Zhang J., Gao T.T., Zhou G.W. (2018). Shape-controlled synthesis of Ni-CeO_2_@PANI nanocomposites and their synergetic effects on supercapacitors. Chem. Eng. J..

[54] Lin C., Gong F., Yang Z., Zhao X., Li Y., Zeng C., Li J., Guo S. (2019). Core-shell structured HMX@polydopamine energetic microspheres: Synergistically enhanced mechanical, thermal, and safety performances. Polymers.

[55] He G., Li X., Bai L., Meng L., Dai Y., Sun Y., Zeng C., Yang Z., Yang G. (2020). Multilevel core-shell strategies for improving mechanical properties of energetic polymeric composites by the “grafting-from” route. Compos. Part B Eng..

[56] Postma A., Yan Y., Wang Y., Zelikin A.N., Tjipto E., Caruso F. (2009). Self-polymerization of dopamine as a versatile and robust technique to prepare polymer capsules. Chem. Mater..

[57] Zhang H., Jiao Q., Zhao W., Guo X., Li D., Sun X. (2020). Enhanced crystal stabilities of ε-CL-20 via core-shell structured energetic composites. Appl. Sci..

[58] Zeng C., Yang Z., Zhang J., Li Y., Lin C., He G., Zhao X., Liu S., Gong F. (2019). Enhanced interfacial and mechanical properties of PBX composites via surface modification on energetic crystals. Polymers.

[59] Salaün F., Devaux E., Bourbigot S., Rumeau P. (2009). Influence of process parameters on microcapsules loaded with n-hexadecane prepared by in situ polymerization. Chem. Eng. J..

[60] Yang Z., Ding L., Wu P., Liu Y., Nie F., Huang F. (2015). Fabrication of RDX, HMX and CL-20 based microcapsules via in situ polymerization of melamine–formaldehyde resins with reduced sensitivity. Chem. Eng. J..

[61] Zhang S., Kou K., Zhang J., Jia Q., Xu Y. (2019). Compact energetic crystals@urea-formaldehyde resin micro-composites with evident insensitivity. Compos. Commun..

[62] Jia X., Wang J., Hou C., Tan Y., Zhang Y. (2018). Effective insensitiveness of melamine urea-formaldehyde resin via interfacial polymerization on nitramine explosives. Nanoscale Res. Lett..

[63] Zhang S., Gao Z., Jia Q., Liu N., Zhang J., Kou K. (2020). Fabrication and characterization of surface modified HMX@PANI core-shell composites with enhanced thermal properties and desensitization via in situ polymerization. Appl. Surf. Sci..

[64] Gong F., Zhang J., Ding L., Yang Z., Liu X. (2017). Mussel-inspired coating of energetic crystals: A compact core–shell structure with highly enhanced thermal stability. Chem. Eng. J..

[65] Li Z., Zhao X., Gong F., Lin C., Liu Y., Yang Z., Nie F. (2020). Multilayer deposition of metal–phenolic networks for coating of energetic crystals: Modulated surface structures and highly enhanced thermal stability. ACS Appl. Energy Mater..

[66] Lin C., Zeng C., Wen Y., Gong F., He G., Li Y., Yang Z., Ding L., Li J., Guo S. (2020). Litchi-like core-shell HMX@HPW@PDA microparticles for polymer-bonded energetic composites with low sensitivity and high mechanical properties. ACS Appl. Mater. Interfaces.

[67] Bian G.Z., Guo X.D., Liu K.W., Li F.S. (2014). In-situ crystallization coating HMX by BAMO-THF copolyether. Chin. J. Explos. Propellants.

[68] Nandi A.K., Ghosh M. (2012). Surface coating of cyclotetramethylenetetranitramine (HMX) crystals with the insensitive high explosive 1,3,5-triamino-2,4,6-trinitrobenzene (TATB). Cent. Eur. J. Energ. Mater..

[69] Dong L.Y., Sheng D.L., Chen L.K., Zhu Y.H., Wang P.Y., Liu L.J. (2016). In-situ coating of TATB on CL-20. Initiat. Pyrotech..

[70] Tan L., Lu X., Liu N., Yan Q.L. (2021). Further enhancing thermal stability of thermostable energetic derivatives of dibenzotetraazapentene by polydopamine/graphene oxide coating. Appl. Surf. Sci..

[71] Zeng G.Y., Nie F.D., Liu L., Chen J., Huang H. (2011). In-situ crystallization coating HMX by polyurethane. Chin. J. Energ. Mater..

[72] Huang H.J., Yang P., Huang H., Li J.S. (2007). Study on HMX coated by in-situ polymerization. Chin. J. Explos. Propellants.

[73] Blank W.J., Layman R.E. (1979). Surfactant-Free Polymer Emulsion Coating Composition and Method for Preparing Same. U.S. Patent.

[74] Bayat Y., Zarandi M., Zarei M.A., Soleyman R., Zeynali V. (2014). A novel approach for preparation of CL-20 nanoparticles by microemulsion method. J. Mol. Liq..

[75] Guo Z.L., Liu J., Li Y.Y., Lin H.F., Wang H., Tam K.C., Liu G.Y. (2021). Effects of dispersion techniques on the emulsion polymerization of methyl methacrylate. Colloid Polym. Sci..

[76] Li Y., Yang Z., Zhang J., Pan L., Ding L., Tian X., Zheng X., Gong F. (2017). Fabrication and characterization of HMX@TPEE energetic microspheres with reduced sensitivity and superior toughness properties. Compos. Sci. Technol..

[77] Liao S.R., Luo Y.J., Sun J., Tan H.M. (2012). Preparation of WPU-g-SAN and its coating on HNIW. Chin. J. Energ. Mater..

[78] Wang J., An C., Ye B., Xu R., Liu Q., Wang J., Dong J. (2020). CL-20/CAB energetic composite microspheres prepared by premix membrane emulsification. AIP Adv..

[79] Jia X., Xu L., Hu Y., Li C., Geng X., Guo H., Liu X., Tan Y., Wang J. (2020). Preparation of agglomeration-free composite energetic microspheres taking PMMA-PVA with honeycomb structure as template via the molecular collaborative self-assembly. J. Energ. Mater..

[80] Kim K.J., Kim H.S. (2005). Coating of energetic materials using crystallization. Chem. Eng. Technol..

[81] Kim K.J., Jung J.W. (2011). Effect of supersaturation on the morphology of coated surface in coating by solution crystallization. Ind. Eng. Chem. Res..

[82] Kim K.J., Kim H.S. (2008). Agglomeration of NTO on the surface of HMX particles in water-NMP solvent. Cryst. Res. Technol..

[83] Reinhard V., Willard R.F., David L.B. (2007). Particle formation in spray drying. J. Aerosol Sci..

[84] Huang C., Liu J., Ding L., Wang D., Yang Z., Nie F. (2017). Facile fabrication of nanoparticles stacked 2,6-diamino-3,5-dinitropyrazine-1-oxide (LLM-105) sub-microspheres via electrospray deposition. Propellants Explos. Pyrotech..

[85] Ye B.Y., An C.W., Wang J.Y., Geng X.H. (2017). Formation and properties of HMX-based microspheres via spray drying. RSC Adv..

[86] Qiu H., Stepanov V., Di Stasio A.R., Chou T., Lee W.Y. (2011). RDX-based nanocomposite microparticles for significantly reduced shock sensitivity. J. Hazard. Mater..

[87] Zhang X.X., Yang Z.J., Nie F., Yan Q.L. (2020). Recent advances on the crystallization engineering of energetic materials. Energetic Mater. Front..

[88] Ma Z., Gao B., Wu P., Shi J., Qiao Z., Yang Z., Yang G., Huang B., Nie F. (2015). Facile, continuous and large-scale production of core–shell HMX@TATB composites with superior mechanical properties by a spray-drying process. RSC Adv..

[89] Yang Y., Li X., Zhao Y., Han Y., Sun Y., Wang J. (2021). Preparation and characterization of core–shell structured FOX-7/F2602 PBX with improved thermal stability and reduced sensitivity. AIP Adv..

[90] Lobry E., Berthe J.E., Hübner J., Schnell F., Spitzer D. (2021). Tuning the oxygen balance of energetic composites: Crystallization of ADN/secondary explosives mixtures by spray flash evaporation. Propellants Explos. Pyrotech..

[91] He G., Liu J., Gong F., Lin C., Yang Z. (2018). Bioinspired mechanical and thermal conductivity reinforcement of highly explosive-filled polymer composites. Compos. Part A Appl. Sci. Manuf..

[92] Hou C., Zhang Y., Chen Y., Jia X., Tan Y. (2018). Fabrication of ultra-fine TATB/HMX cocrystal using a compound solvent. Propellants Explos. Pyrotech..

[93] Huang B., Hao X., Zhang H., Yang Z., Ma Z., Li H., Nie F., Huang H. (2014). Ultrasonic approach to the synthesis of HMX@TATB core-shell microparticles with improved mechanical sensitivity. Ultrason. Sonochem..

[94] Cocero M.J., Martín Á., Mattea F., Varona S. (2009). Encapsulation and co-precipitation processes with supercritical fluids: Fundamentals and applications. J. Supercrit. Fluids.

[95] He B., Stepanov V., Qiu H., Krasnoperov L.N. (2015). Production and characterization of composite Nano-RDX by RESS co-precipitation. Propellants Explos. Pyrotech..

[96] He W., Liu P.J., He G.Q., Gozin M., Yan Q.L. (2018). Highly reactive metastable intermixed composites (MICs): Preparation and characterization. Adv. Mater..

[97] Zhu Y., Zhou X., Xu J., Ma X., Ye Y., Yang G., Zhang K. (2018). In situ preparation of explosive embedded CuO/Al/CL-20 nanoenergetic composite with enhanced reactivity. Chem. Eng. J..

[98] He W., Tao B., Yang Z., Yang G., Guo X., Liu P.J., Yan Q.L. (2019). Mussel-inspired polydopamine-directed crystal growth of core-shell n-Al@PDA@CuO metastable intermixed composites. Chem. Eng. J..

[99] Zhou X., Zhu Y., Cheng Z.P., Ke X., Shi K.W., Zhang K.L. (2019). Preparation of cyclotrimethylenetrinitramine-copper oxide core-shell particles and their thermal decomposition kinetics. Propellants Explos. Pyrotech..

[100] Wharton R.K., Harding J.A. (1994). A study of some factors that affect the impact sensitiveness of liquids determined using the BAM Fall hammer apparatus. J. Hazard. Mater..

[101] Peterson P.D., Idar D.J. (2005). Microstructural differences between virgin and recycled lots of PBX 9502. Propellants Explos. Pyrotech..

[102] Gao H., Hou X.T., Ke X., Liu J., Hao J., Xiao L., Chen T., Zuo Y.Y., Jiang W. (2017). Effects of nano-HMX on the properties of RDX-CMDB propellant: Higher energy and lower sensitivity. Def. Technol..

[103] Liu T., Geng C., Zheng B., Li S., Luo G. (2017). Encapsulation of cyclotetramethylenetetranitramine (HMX) by electrostatically self-assembled graphene oxide for desensitization. Propellants Explos. Pyrotech..

[104] Willey T.M., Lauderbach L., Gagliardi F., Van Buuren T., Glascoe E.A., Tringe J.W., Lee J.R.I., Springer H.K., Ilavsky J. (2015). Mesoscale evolution of voids and microstructural changes in HMX-based explosives during heating through the β-δ phase transition. J. Appl. Phys..

[105] Gong F., Yang Z., Qian W., Liu Y., Zhang J., Ding L., Lin C., Zeng C., Yan Q. (2019). Kinetics for inhibited polymorphic transition of HMX crystal after strong surface confinement. J. Phys. Chem. C.

[106] Kubat J., Rigdahl M., Welander M. (1990). Characterization of interfacial interactions in high density polyethylene filled with glass spheres using dynamic-mechanical analysis. J. Appl. Polym. Sci..

[107] Boddu V.M., Viswanath R.S., Ghosh R.K., Damavarapu R. (2010). 2,4,6-Triamino-1,3,5-trinitrobenzene (TATB) and TATB-based formulations—A review. J. Hazard. Mater..

[108] Lin C., Liu J., Huang Z., Gong F., Li Y., Pan L., Zhang J., Liu S. (2015). Enhancement of creep properties of TATB-based polymer-bonded explosive using styrene copolymer. Propellants Explos. Pyrotech..

[109] Palmas P., Botzanowski T., Guerain M., Forzy A., Bruneton E., Delrio G. (2016). Size determination of porosity inclusions in an organic solid material by (1)H NMR diffusion and SEM-FIB experiments: The TATB case. J. Phys. Chem. B.

[110] Zeng C., Lin C., Zhang J., Liu J., He G., Li Y., Liu S., Gong F., Yang Z. (2019). Grafting hyperbranched polyester on the energetic crystals: Enhanced mechanical properties in highly-loaded polymer based composites. Compos. Sci. Technol..

[111] Simpson R.L., Urtiew P.A., Ornellas D.L., Moody G.L., Scribner K.J., Hoffman D.M. (1997). CL-20 performance exceeds that of HMX and its sensitivity is moderate. Propellants Explos. Pyrotech..

[112] Li J., Brill T.B. (2007). Kinetics of solid polymorphic phase transitions of CL-20. Propellants Explos. Pyrotech..

[113] Ghosh M., Venkatesan V., Mandave S., Banerjee S., Sikder N., Sikder A.K., Bhattacharya B. (2014). Probing crystal growth of ε- and α-CL-20 polymorphs via metastable phase transition using microscopy and vibrational spectroscopy. Cryst. Growth Des..

[114] Zhang P., Xu J.J., Guo X.Y., Jiao Q.J., Zhang J.Y. (2014). Effect of addictives on polymorphic transition of ε-CL-20 in castable systems. J. Therm. Anal. Calorim..

[115] Zheng X., Yu S., Wen W., Wen Y., Wang P., Lan L., Dai X., Han Y., Li J., Li Y. (2018). Sensitivity and phase transition of heated ϵ-CL-20 in drop-weight impact test. Propellants Explos. Pyrotech..

[116] Pan Q., Zhang H.L., Guo X.Y., Zhao W.J., Fan X.K. (2021). Effects of different coatings on the crystal transformation of β-HNIW. J. Cryst. Growth.

[117] Wang Y.Q., Li X., Chen S.S., Ma X., Yu Z.Y., Jin S.H., Li L.J., Chen Y. (2017). Preparation and characterization of cyclotrimethylenetrinitramine (RDX) with reduced sensitivity. Materials.

[118] Jia X., Cao Q., Guo W., Li C., Shen J., Geng X., Wang J., Hou C. (2019). Synthesis, thermolysis, and solid spherical of RDX/PMMA energetic composite materials. J. Mater. Sci. Mater. Electron..

[119] Sun J., Huang H., Zhang Y., Zheng M.X., Liu J.L. (2006). In-situ coating of TATB on HMX. Chin. J. Energ. Mater..

[120] Yan Q.L., Gozin M., Zhao F.Q., Cohen A., Pang S.P. (2016). High energetic compositions based on functionalized carbon nanomaterials. Nanoscale.

[121] Li Y., Yang Z., Liu J., Lin C., Zhang J., Zheng X. (2019). Enhancing fracture toughness of polymer-based functional energetic composites by filling nano-graphene in matrix. Polym. Compos..

